# A Global-Relationship Dissimilarity Measure for the* k*-Modes Clustering Algorithm

**DOI:** 10.1155/2017/3691316

**Published:** 2017-03-28

**Authors:** Hongfang Zhou, Yihui Zhang, Yibin Liu

**Affiliations:** School of Computer Science and Engineering, Xi'an University of Technology, Xi'an 710048, China

## Abstract

The* k*-modes clustering algorithm has been widely used to cluster categorical data. In this paper, we firstly analyzed the* k*-modes algorithm and its dissimilarity measure. Based on this, we then proposed a novel dissimilarity measure, which is named as GRD. GRD considers not only the relationships between the object and all cluster modes but also the differences of different attributes. Finally the experiments were made on four real data sets from UCI. And the corresponding results show that GRD achieves better performance than two existing dissimilarity measures used in* k*-modes and Cao's algorithms.

## 1. Introduction

Clustering is an important technique in data mining, and its main task is to group the given data based on some similarity/dissimilarity measures [1]. Most clustering techniques use distances largely to measure the dissimilarity between different objects [2–4]. However, these methods work only on the data sets with numeric attributes, which limits their uses in solving categorical data clustering problems [5].

Some researchers have made great efforts to quantize relationships among different categorical attributes. Guha et al. [6] proposed a hierarchical clustering method termed ROCK, which can measure the similarity between a pair of objects [7]. In ROCK, the number of* Link* is computed as the number of common neighbors between two objects [8]. However, the following two deficiencies still exist: (1) two involved parameters (*θ*, *k*) must be assigned in advance and (2) the mass calculation is involved [9]. For these reasons, some researchers have generated some new algorithms like QROCK [10], DNNS [11], and GE-ROCK [12] to modify or improve the ROCK algorithm. To remove the numeric-only limitation of* k*-means algorithm, Huang et al. [13, 14] proposed the* k*-modes algorithm, which extends the* k*-means algorithm by using (1) a simple matching dissimilarity measure for categorical attributes; (2) modes in place of means for clustering; and (3) a frequency-related strategy to update modes to minimize the clustering costs [15]. In fact, the idea of simple matching has been used in many clustering algorithms, such as fuzzy* k*-modes algorithm [16], fuzzy* k*-modes algorithm with fuzzy centroid [17], and* k*-prototype algorithm [14]. However, simple matching often results in some low intradissimilarity clusters [18] and disregards of the dissimilarity hidden between the categorical values [19].

In this paper, a Global-Relationship Dissimilarity (GRD) measure for the* k*-modes clustering algorithm is proposed. This dissimilarity measure considers not only the relationships between the object and all cluster modes but also the differences of various attributes instead of simple matching. The clustering effectiveness of* k*-modes based on GRD (KBGRD) is demonstrated on four standard data sets from the UCI Machine Learning Repository [20].

The remainder of this paper is organized as follows: a detailed review of the dissimilarity measure used in* k*-modes is presented and analyzed in Section 2. In Section 3, the new dissimilarity measure GRD is proposed.  Section 4 describes the details of KBGRD algorithm.  Section 5 illustrates the performance and stability of KBGRD. Finally, a concluding remark is given in Section 6.

## 2. Related Works

### 2.1. Categorical Data

As is known to all, the structural data can be stored in a table, where each row represents a fact about an object. And the practical data usually contains categorical attributes [21]. We firstly define the term “data set” [22].

**Definition 1 (data set).**

A data set information system can be expressed as a quadruple IS = {*U*, *A*, *V*, *f*}, which is satisfied with

1. *U* = {*x*_1_, *x*_2_,…, *x*_*n*_} is a nonempty set of *n* data objects, which is named as a universe;
2. *A* = {*a*_1_, *a*_2_,…, *a*_*m*_} is a nonempty set of *m* categorical attributes;
3. *V* is the union of all attribute domains, that is, *V* = ⋃_*j*=1_^*m*^*V*_*a*_*j*__, where *V*_*a*_*j*__ = {*a*_*j*_^(1)^, *a*_*j*_^(2)^,…, *a*_*j*_^(*n*_*j*_)^} is the value domain of attribute *a*_*j*_, and it is finite and unordered; *n*_*j*_ is the number of categories of attribute *a*_*j*_ for 1 ≤ *j* ≤ *m*;
4. *f* is a mapping function *U* × *A* → *V* which can be formally expressed as (∀*x*)(∀*y*)((*x* ∈ *U*)∧(*y* ∈ *A*) → *f*(*x*, *y*) ∈ *V*_*a*_*j*__)  (*j* = 1,2,…, *m*).

### 2.2. *k*-Modes Dissimilarity Measure

The* k*-modes clustering algorithm is an improvement of the* k*-means algorithm [4] by using a simple dissimilarity measure for categorical data. And it adopts a frequency-related strategy to update modes in the clustering to minimize the clustering costs. These extensions have excluded the numeric-only limitation existed in* k*-means algorithm and enable the clustering process to be used on large-size categorical data sets from real world database [22].

**Definition 2 .**

Let IS = {*U*, *A*, *V*, *f*} be a categorical data set information system which is defined in Definition 1 and *a*_*j*_ ∈ *A*. For any object *x*_*i*_ ∈ *U* and cluster mode *z*_*l*_ for 1 ≤ *l* ≤ *k*, Dis_0_(*z*_*l*_, *x*_*i*_) is the simple matching dissimilarity measure between object *x*_*i*_ and the mode *z*_*l*_ of the *l*th cluster which is defined as follows:

(1) $$\begin{array}{c} Dis_{0}\left(\;z_{l},x_{i}\;\right)=\overset{m}{\underset{j=1}{\sum }}\delta^{a_{j}}\left(\;z_{l},x_{i}\;\right) \end{array}$$

In (1), *δ*^*a*_*j*_^(*z*_*l*_, *x*_*i*_) can be expressed as *δ*^*a*_*j*_^(*z*_*l*_, *x*_*i*_) = {1, *f*(*z*_*l*_, *a*_*j*_) ≠ *f*(*x*_*i*_, *a*_*j*_); 0, *f*(*z*_*l*_, *a*_*j*_) = *f*(*x*_*i*_, *a*_*j*_)}.

There are nine objects {*x*_1_, *x*_2_, …, *x*_9_} with four attributes {*A*_1_, *A*_2_, *A*_3_, *A*_4_} and three initial cluster modes as shown in Table 1. For determining the appropriate cluster of *x*_1_, it is required to compute the dissimilarity of *x*_1_ and the three cluster modes. According to (1), Dis_0_(*c*_1_, *x*_1_) = Dis_0_(*c*_2_, *x*_1_) = Dis_0_(*c*_3_, *x*_1_) = 1. Therefore, it is impossible to determine exactly to which cluster the object *x*_1_ should be assigned.

The dissimilarity between an object and a cluster mode should consider the relationships between the object and all cluster modes as well as the differences of various attributes. When the* k*-modes dissimilarity measure is computing dissimilarity of a certain attribute, it only simply matches this object with this mode and ignores the differences of various attributes. Such as attribute “A4” in Table 1, almost all of objects and cluster modes is “E”; “A4” should contribute more to dissimilarity than other attributes. However, the* k*-modes dissimilarity treats all attributes equally.

## 3. Global-Relationship Dissimilarity Measure

**Definition 3 .**

Let IS = {*U*, *A*, *V*, *f*} be a categorical data set information system which is defined in Definition 1 and *a*_*j*_ ∈ *A*. For any object *x*_*i*_ ∈ *U* and cluster mode *z*_*l*_ for 1 ≤ *l* ≤ *k*, Dis(*z*_*l*_, *x*_*i*_) is the new dissimilarity measure between object *x*_*i*_ and the mode *z*_*l*_ of the *l*th cluster which is defined as

(2) $$\begin{array}{c} Dis\left(\;z_{l},x_{i}\;\right)=1-\frac{Sim\left(z_{l},x_{i}\right)}{m}. \end{array}$$

In (2), *m* is the dimension number of data set and the similarity function Sim(*z*_*l*_, *x*_*i*_) is defined as follows:

(3) $$\begin{array}{c} Sim\left(\;z_{l},x_{i}\;\right)=\overset{m}{\underset{j=1}{\sum }}\varphi^{a_{j}}\left(\;z_{l},x_{i}\;\right), \end{array}$$

subject to

(4) $$\begin{array}{c} \varphi^{a_{j}}\left(\;z_{l},x_{i}\;\right)=\left\{\begin{array}{cc} 1-\frac{S-1}{k}, & f\left(z_{l},a_{j}\right)=f\left(x_{i},a_{j}\right)\\ 0, & f\left(z_{l},a_{j}\right)\neq f\left(x_{i},a_{j}\right), \end{array}\right. \end{array}$$

where *k* is the number of cluster modes, and

(5) $$\begin{array}{c} S=\overset{k}{\underset{l=1}{\sum }}s^{a_{j}}\left(\;z_{l},x_{i}\;\right); \end{array}$$

here *s*^*a*_*j*_^(*z*_*l*_, *x*_*i*_) is satisfied with

(6) $$\begin{array}{c} s^{a_{j}}\left(\;z_{l},x_{i}\;\right)=\left\{\begin{array}{cc} 1, & f\left(z_{l},a_{j}\right)=f\left(x_{i},a_{j}\right)\\ 0, & f\left(z_{l},a_{j}\right)\neq f\left(x_{i},a_{j}\right). \end{array}\right. \end{array}$$

As shown in Table 1, it is required to compute the dissimilarity of *x*_1_ with three cluster modes for determining which cluster *x*_1_ should be assigned to. According to (2)–(6), the following three ones can be got:

1. Dis(*c*_1_, *x*_1_) = 1 − (1/4)(1 − (3 − 1)/3 + 0 + 1 − (2 − 1)/3 + 1 − (3 − 1)/3) = 8/12.
2. Dis(*c*_2_, *x*_1_) = 1 − (1/4)(1 − (3 − 1)/3 + 1 − (1 − 1)/3 + 0 + 1 − (3 − 1)/3) = 7/12.
3. Dis(*c*_3_, *x*_1_) = 1 − (1/4)(1 − (3 − 1)/3 + 0 + 1 − (2 − 1)/3 + 1 − (3 − 1)/3) = 8/12.

Hence, *x*_1_ can be assigned to cluster “2” definitely.

## 4. KBGRD Algorithm

In this section, we give the concrete procedure of the* k*-modes based on GRD (KBGRD) algorithm. In addition, the computational complexity of KBGRD is analyzed.

### 4.1. KBGRD Algorithm Description

**Definition 4 .**

Let IS = {*U*, *A*, *V*, *f*} be a categorical data set information system which is defined in Definition 1 and *a*_*j*_ ∈ *A*. The* k*-modes algorithm uses the* k*-means paradigm to cluster categorical data. The objective function of the* k*-modes algorithm is defined as follows:

(7) $$\begin{array}{c} F\left(\;W,Z\;\right)=\overset{k}{\underset{l=1}{\sum }}\overset{n}{\underset{i=1}{\sum }}w_{li}Dis\left(\;z_{l},x_{i}\;\right). \end{array}$$

In (7), {*w*_*li*_ ∈ {0,1}, 1 ≤ *l* ≤ *k*, 1 ≤ *i* ≤ *n*; ∑_*l*=1_^*k*^*w*_*li*_ = 1, 1 ≤ *i* ≤ *n*; 0 < ∑_*i*=1_^*n*^*w*_*li*_ < *n*, 1 ≤ *l* ≤ *k*}. Here *k*(≤*n*) is a known cluster number; *W* = [*w*_*li*_] is a* k*-by-*n*{0,1} matrix; *w*_*li*_ is a binary variable and indicates whether object *x*_*i*_ belongs to the *l*th cluster; *w*_*li*_ = 1 if *x*_*i*_ belongs to the *l*th cluster and 0 otherwise; *Z* = [*z*_1_, *z*_2_,…, *z*_*k*_]; and *z*_*l*_ is the *l*th cluster mode with categorical attributes *a*_1_, *a*_2_, …, *a*_*m*_.

### 4.2. Update and Convergence Analysis

The steps of the KBGRD algorithm are presented below. Here *Z*^(*t*)^ and *W*^(*t*)^ denote cluster modes and membership matrix at *t*th iteration, respectively.

1. Randomly select *k* distinct objects from *U* as initial mode *Z*^(1)^ = [*z*_1_, *z*_2_,…, *z*_*k*_]. Determine *W*^(1)^ such that *F*(*W*^(1)^, *Z*^(1)^) is minimized according to (8). Set *t* = 1.
2. Determine *Z*^(*t*+1)^ such that *F*(*W*^(*t*)^, *Z*^(*t*+1)^) is minimized according to (9). If *F*(*W*^(*t*)^, *Z*^(*t*+1)^) = = *F*(*W*^(*t*)^, *Z*^(*t*)^), then stop; otherwise, go to step (3).
3. Determine *W*^(*t*+1)^ such that *F*(*W*^(*t*+1)^, *Z*^(*t*+1)^) is minimized according to (8). If *F*(*W*^(*t*+1)^, *Z*^(*t*+1)^) = = *F*(*W*^(*t*)^, *Z*^(*t*+1)^), then stop; otherwise, set *t* = *t* + 1 and go to step (2).

In each iteration, *W* and *Z* are updated by the following formulae.

When *Z* is given, *W* is updated by (8) for 1 ≤ *i* ≤ *n* and 1 ≤ *l* ≤ *k*.

(8) $$\begin{array}{c} w_{li}=\left\{\begin{array}{cc} 1, & Dis\left({\hat{z}}_{l},x_{i}\right)\leq Dis\left({\hat{z}}_{h},x_{i}\right),\;\;1\leq h\leq k\\ 0, & otherwise. \end{array}\right. \end{array}$$

And when *W* is given, *Z* is updated as follows:

(9) $$\begin{array}{c} f\left(\;z_{l},a_{j}\;\right)=a_{j}^{\left(r\right)}\in V_{a_{j}}, \end{array}$$

where ∑_*i*=1,*x*_*ij*_=*a*_*j*_^(*r*)^_^*n*^*w*_*li*_*φ*^*a*_*j*_^(*z*_*l*_, *x*_*i*_) ≥ ∑_*i*=1,*x*_*ij*_=*a*_*j*_^(*h*)^_^*n*^*w*_*li*_*φ*^*a*_*j*_^(*z*_*l*_, *x*_*i*_), 1 ≤ *h* ≤ *n*_*j*_. Here, *V*_*a*_*j*__ = {*a*_*j*_^(1)^, *a*_*j*_^(2)^,…, *a*_*j*_^(*n*_*j*_)^}; *n*_*j*_ is the number of categorical of attribute *a*_*j*_ for 1 ≤ *j* ≤ *m*.

Now we consider the convergence of the KBGRD algorithm.

**Theorem 5 .**

$F(W,\hat{Z})$ is minimized when $Z=\hat{Z}$ and *W* is updated by (8).

**Proof**

For a given *Z*, we have $F(W,\hat{Z})=\sum_{l=1}^{k}\sum_{i=1}^{n}w_{li}Dis(z_{l},x_{i})$. The updating method of *W* is computing the minimized dissimilarity between objects and modes according to (8), and the dissimilarities of objects and modes are independent. So *W* is updated by (8) such that $F(W,\hat{Z})$ is minimized.

**Theorem 6 .**

$F(\hat{W},Z)$ is minimized when $W=\hat{W}$ and *Z* is updated by (9).

**Proof**

For a given *W*, we have

(10) FW^,Z∑l=1k ∑i=1nwliDiszl,xi=∑l=1k ∑i=1nwli1−1mSimzl,xi=∑l=1k ∑i=1nwli−1m∑l=1k ∑i=1nwliSimzl,xi=∑l=1k ∑i=1nwli−1m∑l=1k ∑i=1n ∑j=1mwliφajzl,xi=∑l=1k ∑i=1nwli−1m∑l=1k ∑j=1m ∑i=1nwliφajzl,xi=∑l=1k ∑i=1nwli−1m∑l=1k ∑j=1mϕli,

where *ϕ*_*li*_ = ∑_*i*=1_^*n*^*w*_*li*_*φ*^*a*_*j*_^(*z*_*l*_, *x*_*i*_). Note that all inner sums *ϕ*_*li*_ are nonnegative and independent. Then minimizing $F(\hat{W},Z)$ is equivalent to maximizing each inner sum. When *z*_*l*_ = *a*_*j*_^(*r*)^, according to (9), *ϕ*_*li*_ is maximized. So *Z* is updated by (9) such that $F(\hat{W},Z)$ is minimized.

**Theorem 7 .**

The KBGRD algorithm converges in a finite number of iterations.

**Proof**

Firstly, we note that there are only a finite number (*N* = ∏_*j*=1_^*m*^*n*_*j*_) of potential cluster mode. There are *k*^*N*^possible kinds for *k* cluster modes; it is a finite number too.

Secondly, each possible mode appears at most once in the iteration process of KBGRD algorithm. If not, there exist *t*_1_, *t*_2_ (*t*_1_ < *t*_2_) such that *Z*^(*t*_1_)^ = *Z*^(*t*_2_)^. According to Theorem 6, a given *Z* can obtain a certain *W*, that is, *Z*^(*t*_1_)^⇒*W*^(*t*_1_)^, *Z*^(*t*_2_)^⇒*W*^(*t*_2_)^. When *Z*^(*t*_1_)^ = *Z*^(*t*_2_)^, we have *W*^(*t*_1_)^ = *W*^(*t*_2_)^, that is, in the iteration of algorithm, occurring *F*(*W*^(*t*_1_)^, *Z*^(*t*_1_)^) = *F*(*W*^(*t*_1_)^, *Z*^(*t*_2_)^) = *F*(*W*^(*t*_2_)^, *Z*^(*t*_2_)^) at *t*_1_ < *t*_2_. However, if *F*(*W*^(*t*)^, *Z*^(*t*+1)^) = *F*(*W*^(*t*)^, *Z*^(*t*)^) or (*W*^(*t*+1)^, *Z*^(*t*+1)^) = *F*(*W*^(*t*)^, *Z*^(*t*+1)^), algorithm is stopped according to steps (2) and (3) of the KBGRD algorithm, that is, *F*(*W*^(*t*_1_)^, *Z*^(*t*_1_)^) = *F*(*W*^(*t*_1_)^, *Z*^(*t*_2_)^) = *F*(*W*^(*t*_2_)^, *Z*^(*t*_2_)^) never occurs.

So the KBGRD algorithm converges in a finite number of iterations.

### 4.3. Pseudocodes and Complexity Analysis

The pseudocodes of KBGRD algorithm are presented in Pseudocode 1.

The major function of subfunction Cluster() is computing the dissimilarity between object and cluster mode and classifying the objects into the clusters whose dissimilarity is the minimum. The function of subfunction Fun() is computing the value of objective function.

In fact, main function is a controller, which controls the iterations of algorithm. We first choose *k* distinct objects as initial modes. Line 2 is the initialization of cluster; Line 3 computes original cluster result and “new Dissimilarity.” Lines 4–9 are to iteratively update modes and clusters. And when “new Dissimilarity” is invariant, the iteration stops.

Referring to the pseudocodes as shown in Pseudocode 1, the computational complexity of KBGRD algorithm is analyzed as follows. We only consider the major computational steps.

We firstly consider the computational complexity of two subfunctions. The computational complexity for computing the dissimilarity is *O*(*k* · *n* · *m*), where *k* is the number of modes,* n* is the number of objects in data set, and *m* is the dimension of data set. The computational complexity for assigning the *i*th object into the* l*th cluster is *O*(*k* · *n*). So the computational complexity for updating all clusters is *O*(*k* · *n* · (*m* + 1)), that is, *O*(*k* · *n* · *m*). The computational complexity of computing objective function is *O*(*k* · *n* · *m*).

Suppose that the iteration time is *t* and the whole computational cost of KBGRD algorithm is *t*(*O*(*k* · *n* · *m*) + *O*(*k* · *n* · *m*)) = 2*O*(*t* · *k* · *n* · *m*), that is, *O*(*t* · *k* · *n* · *m*). This shows that the computational cost is linearly scalable with the number of objects, the number of attributes, and the number of clusters.

## 5. Experimental Analysis

### 5.1. Experimental Environment and Evaluation Indexes

The experiments are conducted on a PC with an Intel i3 processor and 4 G byte memory running the Windows 7 operating system. All algorithms are coded by JAVA on Eclipse.

To evaluate the efficiency of clustering algorithm, the evaluation indexes* Accuracy (AC)* and* RandIndex* are employed in the experiments.

Let *C* = {*C*_1_, *C*_2_, *C*_3_} be the set of three classes in the data set and *C*′ = {*C*_1_′, *C*_2_′, *C*_3_′} be the set of three clusters generated by the clustering algorithm. Given a pair of objects (*X*_*i*_, *X*_*j*_) in the data set, we refer to it as

1. *a* if both objects belong to the same cluster in *C* and the same cluster in *C*′;
2. *b* if the two objects belong to the same cluster in *C* and two different clusters in *C*′;
3. *c* if the two objects belong to two different clusters in *C* and to the same cluster in *C*′;
4. *d* if both objects belong to two different clusters in *C* and two different clusters in *C*′.

Let *S*_1_, *S*_2_, *S*_3_, and *S*_4_ be the number of* a*,* b*,* c*, and* d*,* RandIndex* [23] is defined as follows:

(11) $$\begin{array}{c} RandIndex=\frac{S_{1}+S_{4}}{S_{1}+S_{2}+S_{3}+S_{4}}. \end{array}$$

*Accuracy (AC)* is defined as follows:

(12) $$\begin{array}{c} AC=\frac{\sum_{i=1}^{k}a_{i}}{n}, \end{array}$$

where *k* is the number of clusters,* n* is the number of objects, and *a*_*i*_ is the number of objects that are correctly assigned to the cluster *C*_*i*_ (1 ≤ *i* ≤ *k*).

Four categorical data sets from the UCI Machine Learning Repository are used to evaluate the clustering performance, including QSAR Biodegradation (QSAR), Chess, Mushroom, and Nursery. The relative information about the data sets is tabulated in Table 2.

### 5.2. Experimental Results and Analysis

In the experiments, we compare KBGRD algorithm with the original* k*-modes and Cao's algorithm [24]. Three algorithms are sequentially run on all data sets. Each algorithm requires the number of modes* (ClusterNum)* as an input parameter. We randomly select distinct* ClusterNum* objects as initial cluster modes. The number of iteration of all algorithms is no more than 500.

Note that there are very few missing values in the Mushroom data set; we use optimal completion strategy to deal with missing values. In the optimal completion strategy, the missing values in data set are viewed as additional variables [25, 26].

Firstly, we set* ClusterNum* as the classes' number of the data set. The average* RandIndex* of ten times' experiments on four data sets for three algorithms is summarized in Table 3. The average* AC* of ten times' experiments on four data sets for three algorithms is summarized in Table 4. As shown in Tables 3 and 4, KBGRD achieves the highest* RandIndex* and* AC*. That is, it performs better than other algorithms under the same conditions.

In real world applications, the number of initial cluster modes is unknown. We evaluated clustering stability by setting different* ClusterNum* (10, 15, 20, 25, 30, and 35) for each data set and used* RandIndex* to evaluate clustering results. The average* RandIndex* of ten times' experiments on four data sets for three algorithms is summarized in Tables 5–8. And the last column shows the average clustering* RandIndex* of each algorithm on six* ClusterNum*. As shown in Tables 5–8, KBGRD achieves the highest* RandIndex*. That is to say, it performs better than other algorithms on four data sets. Additionally, KBGRD has the highest stability compared with other algorithms.

## 6. Conclusion

This paper analyzes the advantages and disadvantages of* k*-modes algorithms for categorical data. Based on this, we propose a novel dissimilarity measure (GRD) for clustering categorical data. This measure is used to improve the performance of the existing* k*-modes algorithm. The computational complexity of KBGRD algorithm has been analyzed which is linear with the number of data objects, attributes, and clusters. We have tested KBGRD algorithm on four real data sets from UCI. Experimental results have shown that KBGRD algorithm is effective and stable in clustering categorical data sets.

## Figures and Tables

**Pseudocode 1:**
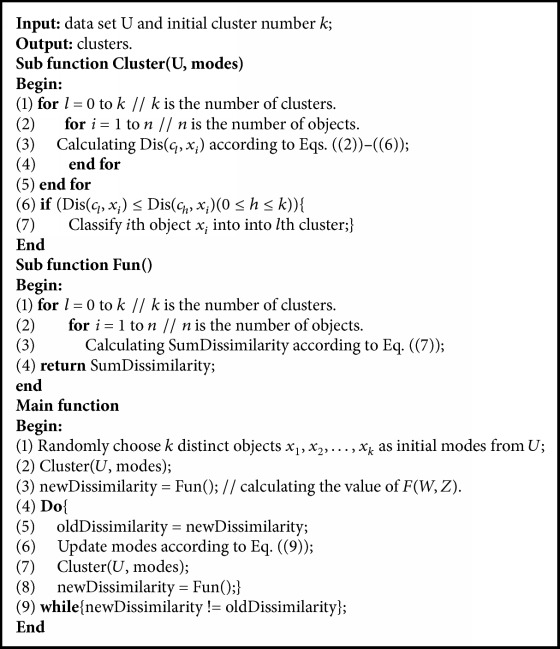
Pseudocodes of KBGRD algorithm.

**Table 1:** An artificial data set.

Objects	*A*_1_	*A*_2_	*A*_3_	*A*_4_
*x*_1_	A	B	A	E
*x*_2_	A	A	B	D
*x*_3_	C	A	A	E
Cluster 1 (*c*_1_)	A	A	A	E
*x*_4_	A	B	B	E
*x*_5_	B	A	C	E
*x*_6_	A	B	C	E
Cluster 2 (*c*_2_)	A	B	C	E
*x*_7_	A	A	A	E
*x*_8_	D	C	B	E
*x*_9_	C	C	A	E
Cluster 3 (*c*_3_)	A	C	A	E

**Table 2:** Data sets.

Data set	Attribute characteristics	#of data objects	# of attributes	# of class	Missing values
QSAR	Integer/real	1055	41	2	No
Chess	Categorical	3196	36	2	No
Mushroom	Categorical	8142	22	2	Yes (very few)
Nursery	Categorical	12960	8	5	No

**Table 3:** Average *RandIndex* on four data sets for three algorithms.

	QSAR	Chess	Mushroom	Nursery
*k*-modes	0.513	0.5102	0.5101	0.6908
Cao's	0.5106	0.5136	0.5251	0.7895
KBGRD	0.5153	0.5229	0.5543	0.7933

**Table 4:** Average AC on four data sets for three algorithms.

	QSAR	Chess	Mushroom	Nursery
*k*-modes	0.5820	0.5720	0.5701	0.4786
Cao's	0.5944	0.5432	0.5895	0.5897
KBGRD	0.6042	0.6073	0.6634	0.5938

**Table 5:** Average *RandIndex* of three algorithms on QSAR data set.

	10	15	20	25	30	35	Average
*k*-modes	0.4613	0.4608	0.4611	0.4596	0.4603	0.4584	0.4603
Cao's	0.4650	0.4610	0.4625	0.4608	0.4611	0.4593	0.4616
KBGRD	0.4658	0.4634	0.4628	0.4612	0.4620	0.4605	0.4626

**Table 6:** Average *RandIndex* of three algorithms on Chess data set.

	10	15	20	25	30	35	Average
*k*-modes	0.5016	0.5011	0.5008	0.5024	0.5032	0.5027	0.5020
Cao's	0.5041	0.5023	0.5014	0.5064	0.5045	0.5044	0.5039
KBGRD	0.5060	0.5090	0.5073	0.5072	0.5070	0.5075	0.5074

**Table 7:** Average *RandIndex* of three algorithms on Mushroom data set.

	10	15	20	25	30	35	Average
*k*-modes	0.5771	0.5641	0.5611	0.5622	0.5443	0.5404	0.5582
Cao's	0.5925	0.5644	0.5679	0.5790	0.5558	0.5638	0.5706
KBGRD	0.5932	0.5648	0.5731	0.5834	0.5678	0.5730	0.5759

**Table 8:** Average *RandIndex* of three algorithms on Nursery data set.

	10	15	20	25	30	35	Average
*k*-modes	0.6839	0.7061	0.6963	0.6875	0.6815	0.6942	0.6916
Cao's	0.7188	0.7071	0.6956	0.6989	0.6847	0.6982	0.7006
KBGRD	0.7195	0.7073	0.6967	0.7022	0.6957	0.6988	0.7034
